# Comparative Evaluation of Strep A Throat Swab Culture Results Using the RapidFor™ Strep A Rapid Test Kit

**DOI:** 10.3390/children13040454

**Published:** 2026-03-26

**Authors:** Belen Ates, Meryem Cansu Olt, Alper Kacar, Nazmiye Yeni, Eren Guzeloglu, Cigdem Arabaci

**Affiliations:** 1 Department of Pediatrics, Prof. Dr. Cemil Tascioglu City Hospital, University of Health Sciences, 34384 Istanbul, Türkiye; naz_ua_41@hotmail.com (N.Y.); dr.erenguzeloglu@gmail.com (E.G.); 2 Department of Ethics Committee, Prof. Dr. Cemil Tascioglu City Hospital, University of Health Sciences, 34384 Istanbul, Türkiye; cansuolt@gmail.com; 3 Department of Pediatric Emergency, Prof. Dr. Cemil Tascioglu City Hospital, University of Health Sciences, 34384 Istanbul, Türkiye; dralperkacar@gmail.com; 4 Department of Clinical Microbiology, Prof. Dr. Cemil Tascioglu City Hospital, University of Health Sciences, 34384 Istanbul, Türkiye; dr.c.arabaci@hotmail.com

**Keywords:** Group A Streptococcus, pharyngitis, rapid antigen detection test, throat culture, pediatric

## Abstract

**Highlights:**

**What are the main findings?**

- The RapidFor™ Strep A test showed very high sensitivity and specificity in children.
- A negative predictive value of 99.6% supports safe exclusion of Group A Streptococcus (GAS) pharyngitis.

**What are the implications of the main findings?**

- RapidFor™ Strep A enables safe same-visit exclusion of GAS pharyngitis, reducing unnecessary cultures and antibiotic use.
- High agreement with throat culture supports its use as a frontline diagnostic tool, enabling earlier targeted antibiotic therapy and faster prevention of complications.

**Abstract:**

**Background/Objectives:** Group A Streptococcus (GAS) pharyngitis is a frequent cause of morbidity in pediatric populations, which requires timely identification to prevent complications such as acute rheumatic fever. Rapid antigen detection tests (RADTs) are practical alternatives to throat culture. This study evaluates the diagnostic performance of the RapidFor™ Strep A test. **Methods:** This prospective clinical study enrolled 389 pediatric patients aged < 18 years with symptoms suggestive of streptococcal pharyngitis. Two throat swabs were collected from each patient: one for rapid antigen testing with RapidFor™ Strep A and one for culture. Sensitivity, specificity, positive predictive value (PPV), and negative predictive value (NPV) were calculated. **Results:** Throat culture was positive in 95 of 389 patients (24.4%). The RapidFor™ Strep A test demonstrated a sensitivity of 98.95% (95% confidence interval [CI]: 94.28–99.81%) and a specificity of 96.26% (95% CI: 93.43–97.90%). The PPV was 89.52%, and the NPV was 99.65%. Agreement with culture was excellent (κ = 0.919); in particular, false-positive results accounted for 2.8% and false-negative results accounted for 1.05%. Fever was the strongest clinical indicator associated with positive results. **Conclusions:** The RapidFor™ Strep A test showed very high diagnostic accuracy compared with throat culture, including an excellent NPV (99.6%), which supports its reliability for ruling out GAS pharyngitis in pediatric settings. The test is an effective screening tool that facilitates timely antibiotic therapy.

## 1. Introduction

Group A Streptococcus (GAS) is a Gram-positive, extracellular, and spherical β-hemolytic bacterium that is capable of growing on enrichment media for culture [1]. GAS is associated with a wide range of clinical conditions, including acute pharyngitis, sepsis, meningitis, scarlet fever, acute rheumatic fever, glomerulonephritis, necrotizing fasciitis, impetigo, and streptococcal toxic shock syndrome [2]. In pediatric practice, GAS is a key bacterial cause of upper respiratory tract infection, most commonly presenting as acute pharyngitis/tonsillopharyngitis. Acute pharyngitis caused by GAS remains a major source of morbidity in childhood and is one of the leading causes of physician visits in pediatric and adult populations worldwide [3,4]. Although clinical algorithms exist, diagnosis based solely on clinical findings is difficult, and throat cultures and rapid antigen detection tests (RADTs) are frequently used for confirmation [5,6]. Overreliance on clinical findings alone may contribute to inappropriate antibiotic prescribing, highlighting the need for accurate and rapid diagnostic tools to support antimicrobial stewardship.

GAS accounts for up to 30% of children presenting with pharyngitis symptoms [7]. However, asymptomatic GAS carriage in children may complicate diagnostic interpretation, making test accuracy and appropriate clinical correlation particularly important. According to 2024 World Health Organization (WHO) standards, the incidence of GAS pharyngitis in children aged 5 to 14 years is 22.1 per 100 child-years, representing approximately 288.6 million cases globally each year [8]. In pediatric practice, GAS is a key bacterial cause of upper respiratory tract infection, most commonly presenting as acute pharyngitis/tonsillopharyngitis [9]. The primary goal of treating GAS pharyngitis in pediatric patients is to prevent severe complications, including sepsis and acute rheumatic fever. Early treatment also reduces the spread of infection, shortens symptom duration, and lowers the risk of suppurative complications, including cervical lymphadenitis, retropharyngeal abscess, and peritonsillar abscess [9]. RADTs are preferred in many settings because they provide same-day results, reduce unnecessary antibiotic use, and assist clinicians in initiating appropriate therapy promptly [10]. Even though RADTs are widely used, the test platform, epidemiological setting, and patient group can all affect diagnostic performance. Local validation studies are therefore necessary to guarantee dependable application in standard clinical practice.

This prospective study evaluated the diagnostic accuracy of the RapidFor™ Strep A Rapid Test Kit (Vitrosens Biotechnology, Istanbul, Türkiye) in pediatric patients with acute pharyngitis, compared with throat culture. In addition, this study aimed to assess age-specific diagnostic performance and to explore clinical predictors associated with culture positivity in a real-world pediatric outpatient setting.

## 2. Materials and Methods

This prospective clinical study was conducted in the Department of Pediatrics at Prof. Dr. Cemil Tascioglu City Hospital, Istanbul, Türkiye. This study was designed and reported in accordance with the STARD 2015 guidelines for diagnostic accuracy studies. Ethical approval was obtained from the Institutional Ethics Committee of Prof. Dr. Cemil Tascioglu City Hospital, Istanbul, Türkiye. (Ethics Committee Number: E-48670771-514.99-266740643; Date: 27 January 2025) and the Turkish Medicines and Medical Devices Agency (E-68869993-000-5578552). The study was performed between 3 February 2025 and 1 April 2025. Consecutive eligible pediatric patients presenting during the study period were invited to participate in order to minimize selection bias. Enrollment followed the standard clinical procedure applied to all patients presenting with pharyngitis symptoms at our institution; rather, the decision to obtain a throat culture was made independently by the physicians based on clinical assessment. Patients for whom a throat culture had already been ordered were subsequently enrolled and also tested with the RADT, ensuring that study participation did not influence clinical decision making. The kit manufacturer had no role in patient selection or enrollment decisions at any stage. To ensure that this study was conducted impartially, an independent monitor followed the work throughout its duration. The independent monitor was appointed by the hospital research oversight unit to oversee adherence to the protocol; the sponsor had no role in the monitor’s appointment or activities.

Sample size estimation followed Buderer’s method for diagnostic accuracy studies. Assuming a projected prevalence of GAS pharyngitis of 25%, a target sensitivity and specificity of 95%, and a precision of ±5% for the 95% confidence interval (CI), the minimum required sample size was 73 individuals. To account for potential data loss or testing failures, the planned enrollment was approximately 400 individuals. The final cohort consisted of 389 patients, including 95 culture-positive cases, which exceeded the minimum requirement and yielded 95% confidence intervals with satisfactory precision for both sensitivity (width: 5.53%) and specificity (width: 4.47%).

**Figure 1:**
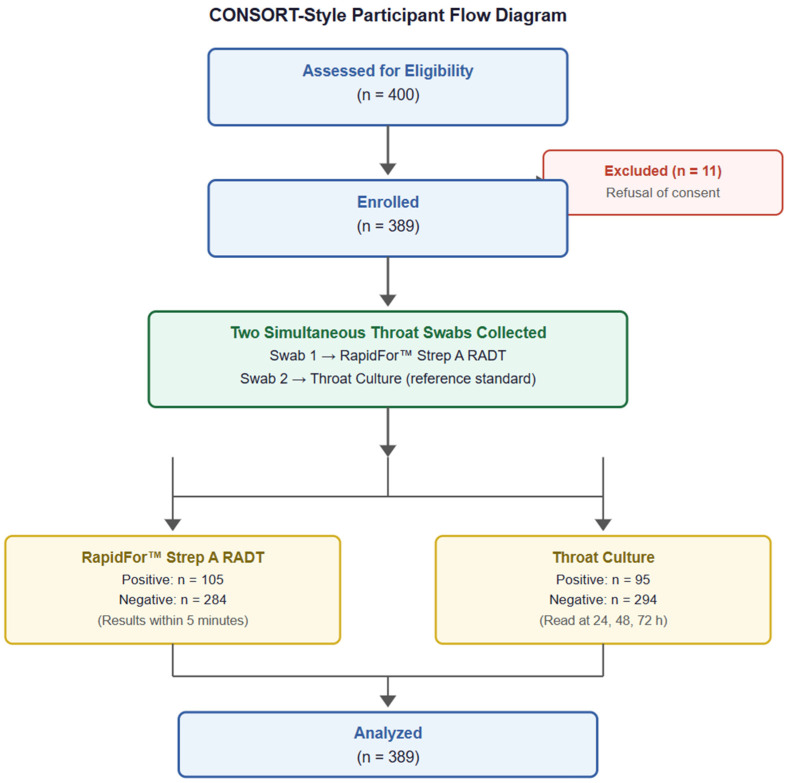
CONSORT-style participant flow diagram.

A total of 389 pediatric patients presenting to the pediatric outpatient clinic with clinical findings suggestive of streptococcal tonsillopharyngitis between February of 2025 and April of 2025 were included. Inclusion criteria were as follows: (1) Age ≤ 18 years. (2) Clinical findings recorded included tonsillar enlargement, tonsillar exudate, oropharyngeal hyperemia, and tender anterior cervical lymphadenopathy as part of the routine physical examination. Oropharyngeal hyperemia was documented descriptively and was not used as an independent diagnostic criterion for GAS infection. (3) Parental or guardian consent, with assent when appropriate. Exclusion criteria were as follows: (1) age > 18 years; (2) current or recent antibiotic use within 7 days of enrollment; (3) known immunodeficiency or immunosuppressive therapy; (4) inability or refusal to provide informed consent; or (5) incomplete sampling or test data. Only symptomatic children with clinically suspected acute pharyngitis were included in this study. Asymptomatic individuals and potential healthy GAS carriers were not evaluated within the study design. A participant flow diagram illustrating enrollment, exclusions, and final sample is provided as Figure 1.

After informed consent, two consecutive throat swabs were collected. Both were collected from the tonsils and posterior pharynx using a standard technique. Contact with the tongue, buccal mucosa, and lips was avoided. One swab was transported to the microbiology laboratory for culture on blood agar, and the second was used for the RapidFor™ Strep A Rapid Test Kit. The rapid test delivers results within 5 min and was recorded by the principal investigator, with each patient assigned a unique study number. Culture results were retrieved from the hospital microbiology system at 24, 48, and 72 h. RADT was performed immediately at the point of care according to the manufacturer’s instructions. The investigators performing the rapid antigen test were blinded to culture results, and microbiology laboratory personnel were blinded to rapid test outcomes. Study data were collected prospectively using unique patient identifiers and kept in a password-protected institutional database accessible only to the study investigators. No external parties, including the kit manufacturer, had access to the data at any point. Data integrity was managed by an independent monitor appointed by the hospital research oversight unit.

In brief, the diagnostic pathway was as follows: upon enrollment, two throat swabs were simultaneously collected from each patient. The first swab was immediately tested using the RapidFor™ Strep A RADT at point-of-care, yielding results within 5 min. The second swab was placed in Stuart transport medium and delivered to the microbiology laboratory within 30 min for standard culture.

Swabs for culture were placed in Stuart transport medium and transferred to the microbiology laboratory. Specimens were transported to the laboratory within 30 min of collection to preserve viability. Specimens were inoculated onto 5% sheep blood agar using the streak plate dilution technique and incubated at 37 °C for 24 h. Plates were re-examined at 48 h (and, when required by routine laboratory workflow, up to 72 h) before being reported as negative. A culture was considered negative if no β-hemolytic colonies consistent with *Streptococcus* spp. were observed at the final reading. Colonies showing β-hemolysis were further evaluated, and identification of Streptococcus pyogenes was confirmed using the Vitek MS automated system (bioMérieux, Paris, France). In specimens with mixed growth, suspected β-hemolytic colonies were subcultured to obtain isolated colonies prior to identification. All culture procedures and identification steps were performed according to the microbiology laboratory’s standard operating procedures and routine quality-control practices.

Demographic characteristics and detailed clinical findings were recorded at presentation, including fever, sore throat, dysphagia, tonsillar enlargement or exudate, oropharyngeal hyperemia, tender anterior cervical lymphadenopathy, cough, rhinorrhea, and duration of symptoms. Recent or current antibiotic use was also documented.

All analyses were conducted using IBM SPSS Statistics version 23 (IBM Corp., Armonk, NY, USA). Frequencies and percentages were analyzed for categorical variables. Normally distributed continuous variables are expressed as mean ± standard deviation (SD), whereas non-normally distributed data are summarized as median with interquartile range (IQR). Group comparisons of categorical variables employed the chi-square test or Fisher’s exact test, when appropriate. Age-stratified comparisons of RADT and culture positivity rates were evaluated using the chi-square test for trends. Given the lower epidemiological prevalence of GAS pharyngitis in children under 3 years of age, additional age stratification (<3 years, 3–5 years, 6–12 years, 13–17 years) was performed to enhance interpretability. The diagnostic performance of the RapidFor™ Strep A Rapid Test Kit—including sensitivity, specificity, positive predictive value (PPV), and negative predictive value (NPV)—was calculated using standard formulas with throat culture as the reference standard. The Wilson score method was used to compute 95% CI values. Throat culture was used as the reference standard for all diagnostic accuracy estimates; a separate clinical reference standard (e.g., incorporating CRP) was not defined within the study design. The Wilson method was selected to provide more accurate interval estimation for proportions close to 0 or 1. Agreement between RADT and culture results was assessed using Cohen’s Kappa coefficient, interpreted according to the Landis and Koch criteria.

We employed multivariable logistic regression analysis to identify independent factors associated with positive culture. Variables were selected a priori based on clinical relevance and supported by univariate screening; the final multivariable model retained variables that met statistical significance (*p* < 0.05) and/or were considered clinically important confounders, and adjusted ORs with 95% CIs were reported. Adjusted odds ratios (ORs) with 95% confidence intervals were reported. No missing data were present for variables included in the regression model. Multicollinearity was assessed using variance inflation factors (VIF), with VIF < 5 considered acceptable. To reduce overfitting, model complexity was limited to maintain an adequate events-per-variable ratio. The Hosmer–Lemeshow goodness-of-fit test was utilized to assess model fit. Model discrimination was evaluated using the area under the receiver operating characteristic curve (AUC). All tests were two-tailed, and a *p*-value < 0.05 was considered statistically significant. All statistical analyses were conducted by study investigators who were unaware of the manufacturer’s claimed performance standards. The absence of independent replication of these results represents a limitation; external validation by more research groups is recommended as a crucial step for future research.

## 3. Results

### Symptom-Based Positivity Rates and Diagnostic Performance of the Rapid Antigen Detection Test

Throat cultures were positive in 95 of 389 patients, yielding a culture positivity rate of 24.4%. The mean age was 7.9 years, the median age was 8.0 years, and the age range was 1.0–18.0 years. The sex distribution was 58% male and 42% female. The age-stratified diagnostic performance of the RADT is summarized in Table 1.

When symptom groups were evaluated as mutually exclusive categories (Table 2), culture positivity was 8.7% (6/69) in patients with fever only and 13.6% (8/59) in those with sore throat only. The highest positivity was observed in patients presenting with both fever and sore throat, with RADT positivity of 47.7% (74/155) and culture positivity of 43.9% (68/155). In patients with other or mixed symptom profiles, culture positivity was 12.3% (13/106). Overall, culture positivity differed significantly across symptom groups (*p* < 0.001). Symptom-group analyses are summarized in Table 2, showing culture positivity and overall RADT accuracy across symptom categories.

Among patients presenting with fever and sore throat, RADT positivity was 47.7% (74/155) and culture positivity was 43.9% (68/155). Patients presenting with fever and rhinorrhea had a culture positivity rate of 20.0% (1/5); however, rhinorrhea was analyzed descriptively and is not considered a classical predictor of GAS pharyngitis. In those with fever and cough, RADT positivity was 13.9% (5/36), and culture positivity was 8.3% (3/36). In the sore throat and cough group, RADT positivity was 25.0% (5/20), and culture positivity was 20.0% (4/20).

Independent predictors of culture positivity are summarized in Table 3. Recent antibiotic use was associated with reduced culture positivity (OR = 0.42, 95% CI: 0.20–0.90, *p* = 0.026). Older age was associated with higher odds of culture positivity (OR 1.11, 95% CI 1.05–1.18; *p* < 0.001), and fever was a significant predictor (OR 2.61, 95% CI 1.38–4.93; *p* = 0.003). Although oropharyngeal hyperemia was frequently observed, it did not demonstrate independent predictive value for culture positivity after multivariable adjustment.

The analysis showed that the RADT had a high sensitivity of 98.95% (95% CI: 94.28–99.81%) and a specificity of 96.26% (95% CI: 93.43–97.90%) when compared with throat culture as the reference. The PPV was 89.52%, and the NPV was 99.65%, based on the 24.4% disease prevalence in the study population.

**Figure 2:**
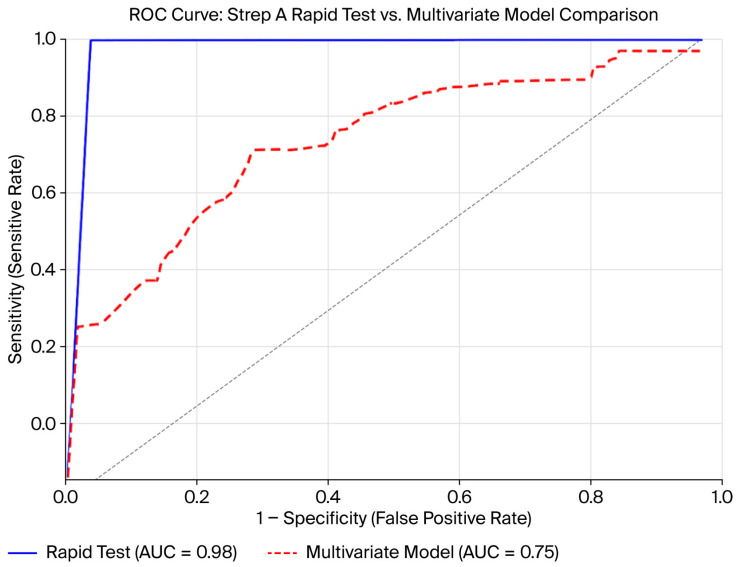
Receiver operating characteristic (ROC) curve comparing the diagnostic accuracy of the RapidFor™ Strep A Rapid Test Kit and the multivariate logistic regression model.

Receiver operating characteristic (ROC) curve analysis was performed to further assess the diagnostic efficacy of the RapidFor™ Strep A Rapid Test Kit. The area under the curve (AUC) was 0.98 (95% CI: 0.96–0.99), indicating excellent discrimination between culture-positive and culture-negative cases. The multivariable logistic regression model that included clinical factors, such as fever, tonsillar exudate, lymphadenopathy, and antibiotic use, produced an AUC of 0.75, demonstrating moderate discriminative ability (Figure 2). The model showed acceptable calibration (Hosmer–Lemeshow *p* = 0.601) and moderate discrimination (AUC = 0.75), indicating that clinical features alone provide limited standalone predictive performance. ROC curves for the clinical model were generated using the model’s predicted culture-positivity probabilities.

Among 389 specimens, 11 (2.8%) produced false-positive results, most occurring in children aged 6–12 years (n = 6, 54.5%) with recent antibiotic exposure or mild tonsillar exudates. The false-positive rate was 3.7% (11/294). Potential explanations include (1) cross-reactivity with Group C or G Streptococcus colonization, (2) transient antigen persistence after recent infection, or (3) nonviable GAS organisms following antibiotic therapy. These results emphasize the importance of clinical correlation when interpreting positive rapid test results, particularly in patients with recent antibiotic use.

There was one false-negative result (0.3%), corresponding to a false-negative rate of 1.05% (1/95). The patient was aged 6–12 years and had received antibiotics shortly before sampling, likely reducing antigen levels below the detection threshold. This finding highlights the need to collect specimens before initiating antibiotics and demonstrates the high overall accuracy of the test in detecting GAS infection.

Performance characteristics are summarized in Table 4. The test demonstrated high sensitivity (98.95%) and specificity (96.26%) and excellent NPV (99.65%), supporting its clinical utility.

## 4. Discussion

In our study, the throat culture positivity rate was 24.4% among pediatric patients with pharyngitis symptoms. A meta-analysis reported that the prevalence of GAS pharyngitis in children usually ranges from 20% to 30%, which is consistent with our findings [11]. The agreement between our data and prior studies suggests that our sample is representative and that the diagnostic methods used were appropriate.

When stratified by age, we observed a clear increase in RADT and culture positivity with advancing age, indicating that GAS pharyngitis is more common in adolescents. Although GAS pharyngitis is uncommon in children under 3 years of age, testing may still occur in clinical practice due to overlapping symptomatology with viral infections. This pattern is supported by the literature. Wi and Choi [12] reported GAS prevalence rates of 19.3% in children aged 3–5 years, 17.6% in those aged 6–10 years, and 27.8% in those aged 11–14 years. Although age categories vary slightly, both datasets show a consistent rise in GAS positivity with age. These findings indicate that school-aged children and adolescents have a higher risk of streptococcal pharyngitis, likely because of greater exposure in academic and social environments. This age-related gradient in positivity further supports the importance of age-stratified diagnostic interpretation in pediatric clinical practice.

Our results also demonstrated that positivity rates for RADTs and cultures differed significantly by symptom profile (*p* < 0.001). Febrile children exhibited substantially higher positivity than those in other symptom groups, indicating that fever and sore throat are strong clinical indicators of streptococcal infection. In a multivariable logistic regression model including age and fever, both variables remained independently associated with culture positivity. The literature similarly reports a significant association between fever, sore throat, and positive test results [13]. The small number of positives among patients with fever and cough or fever and rhinorrhea supports established guidance, in that these symptoms are more closely associated with viral upper respiratory tract infections than GAS [14]. Rhinorrhea was included as a recorded clinical variable for descriptive completeness but was not interpreted as an independent predictor of GAS infection. These findings highlight the importance of fever combined with sore throat in improving diagnostic accuracy for streptococcal pharyngitis. Although oropharyngeal hyperemia was commonly observed, it limits specificity and should not be regarded as an independent diagnostic indicator for GAS infection in the absence of additional clinical symptoms.

Fever emerged as the most reliable clinical predictor of positive results, with significantly higher RADT and culture positivity among febrile patients than among those with other symptoms (*p* < 0.001 for both), consistent with previous studies [15]. After adjusting for confounding variables, our multivariable analysis showed that patients who had recently taken antibiotics within the last month, including penicillin and cephalosporins, had markedly reduced risks of positive RADT and culture results. Although this association was not evident in crude comparisons, the adjusted model clarified the independent impact of recent antibiotic exposure. This finding aligns with studies showing that antibiotic exposure can suppress the growth or detectability of GAS. Prior research involving children similarly reported that those who had taken antibiotics in the preceding month were more likely to test negative despite clinical symptoms [16]. Antibiotic pretreatment can reduce bacterial load in the oropharynx below the detection threshold of throat cultures or RADTs, especially when antibiotics are administered before sample collection, and even modest suppression may lower test sensitivity.

The RADT achieved high sensitivity (98.9%) and specificity (96.3%) compared with culture. Significantly, research independent of commercial input has indicated sensitivities ranging from 70% to 84% for similar RADT technologies [17,18,19], suggesting that our higher estimates may be attributable to the symptom-rich population and standardized single-center settings rather than to the superiority of the test platform. No statistically significant disparities in specificity were noted between our findings and those from independent assessments. In our study, the false-positive rate was 3.7% (11 out of 294 culture-negative instances). The possible causes include persistence of antigen after recent infection, cross-reactivity with non–group A β-hemolytic streptococci, or diagnosis in colonized individuals. manufacturer-specific searches into false positives could not be independently confirmed in our dataset. This comparatively high sensitivity is more plausibly explained by the symptom-enriched cohort (≥2 GAS-compatible symptoms), exclusion of recent antibiotic use, the single-center prospective design with standardized sampling, and potential workflow factors (including blinding), rather than test technology alone. These findings suggest that modern RADTs can achieve diagnostic accuracy comparable to throat cultures when used under optimal clinical conditions. Although it should be noted that it may not be possible to directly extrapolate sensitivity estimates from symptom-enriched, single-center cohorts with a defined pre-test probability to heterogeneous primary-care populations. The results should be interpreted in light of the narrow absolute number of culture-positive cases (n = 95), which also broadens the sensitivity confidence interval. However, the consistently high performance across all age groups and symptom categories increases confidence in the validity of these estimates in our clinical setting. Notably, an NPV of 99.6% underscores the clinical utility of the RADT in excluding infection. A negative result almost certainly indicates the absence of GAS, reducing unnecessary antibiotic use and the need for follow-up cultures. This is especially important in primary care and emergency settings, where timely decisions are essential. A PPV of 89.5% suggests that clinical confirmation may be necessary. These findings align with the literature. In high-prevalence settings, positive results are more likely to reflect true infection, whereas in lower-prevalence populations, careful clinical correlation remains essential. Stewart et al. [20] conducted a meta-analysis reporting aggregated RADT sensitivities and specificities of 86% and 96%, respectively, with improved diagnostic accuracy in pediatric populations.

Several prior studies have evaluated RADT performance with variable results. PPVs ranged from 62.2% to 100%, and NPVs ranged from 89% to 100% across different platforms and populations [21,22,23]. Our results (PPV: 89.52%; NPV: 99.65%) fall within this range, indicating that the RapidFor™ Strep A test provides predictive performance comparable to previously validated RADTs.

Studies have shown that using RADTs before antibiotic therapy is more cost-effective for acute tonsillopharyngitis than direct culture and immediate treatment [23]. The most widely used RADTs are new-generation assays based on lateral migration immunoassays and rapid immune chromatographic methods [24]. A notable strength of our study is that all samples were cultured concurrently with the gold-standard method, while the rapid antigen test was evaluated for false positivity, consistent with the quality standards defined by the Quality Assessment of Diagnostic Accuracy Studies (QUADAS) criteria [25].

The Cohen’s Kappa coefficient between RADT and throat culture was 0.919, indicating almost perfect agreement, consistent with previous studies reporting κ values of 0.848-0.91 [26,27,28].

These findings have important clinical implications. The use of high-performing RADTs in routine pediatric care can improve antibiotic management by reducing unnecessary antibiotic use and antibiotic resistance. The considered test enables same-day treatment decisions in primary care and emergency settings, reduces unnecessary antibiotic use, and facilitates timely therapy to prevent complications such as acute rheumatic fever. Nucleic acid amplification tests (NAATs), including PCR, offer greater analytical sensitivity than culture and are increasingly regarded as the reference standard in GAS diagnostics. In our study, throat culture was used as the sole reference, which introduces an inherent limitation: the sensitivity of culture for GAS ranges from 90 to 95% under optimal laboratory conditions. As a result, some RADT-positive/culture-negative cases labeled as false positives may actually represent true GAS infections that culture failed to detect. This likely results in an underestimation of RADT specificity rather than an overestimation of sensitivity. Future research using NAAT as a concurrent or supplementary reference standard would allow for more accurate classification of conflicting case outcomes.

This study has a few limitations. First, the single-center design was conducted at a tertiary pediatric center in Istanbul, which exclusively enrolled symptomatic children, limiting the generalizability to asymptomatic carriers, adults, or other clinical environments; multicenter validation is necessary. Second, only one commercial test kit was assessed. Third, the study population consisted exclusively of symptomatic patients with clinically suspected GAS pharyngitis. The diagnostic performance of the RADT in asymptomatic individuals or healthy GAS carriers was not assessed. Since asymptomatic carriage may influence specificity estimates, our findings may not be directly generalizable to population-level screening settings. Because enrollment required ≥2 GAS-compatible symptoms, our cohort likely had an increased pre-test probability, which may have inflated performance estimates (particularly PPV) compared with broader primary-care populations, including children with viral symptom profiles. Additionally, the logistic regression model lacked internal validation; to assess model stability, future studies with larger sample sizes should incorporate bootstrapping or cross-validation techniques.

Fourth, although patients with recent antibiotic use were excluded, antibiotic history was self-reported and susceptible to recall bias, which may have resulted in misclassification. Future research should incorporate multicenter designs, cost-effectiveness analyses, and long-term clinical outcome assessments. Future studies should also evaluate RADT performance in asymptomatic carriers and compare molecular diagnostics to further refine testing strategies in pediatric populations. This study took place over two months (February–April 2025), aligning with Istanbul’s peak respiratory season. The short recruitment window might limit the seasonal consistency of the sample and lead to cluster effects from concurrent GAS outbreaks. Diagnostic performance metrics, mainly prevalence-dependent indicators like PPV, could fluctuate during periods of lower epidemiological activity. Future research covering an entire calendar year and including various seasonal periods is crucial to confirm the reliability and broad applicability of the observed diagnostic performance. Additionally, the absence of a molecular reference standard means that some culture-negative RADT-positive results cannot be definitively classified as false positives; a proportion may represent genuine GAS infections below the culture detection threshold.

## 5. Conclusions

The Strep A rapid diagnostic test demonstrated high accuracy compared with the gold-standard culture method. Its strong reliability in terms of negative results (99.6% NPV) indicates that the test can be used safely in clinical practice. However, positive results should be interpreted alongside clinical evaluation and supplemented with additional testing when necessary. The findings support the use of RADTs as effective screening tools in pediatric populations. By identifying potential cases at presentation, clinicians can initiate antibiotic therapy earlier, reducing illness duration and transmissibility. Early treatment may also help to prevent complications such as sepsis and acute rheumatic fever, which are important causes of morbidity and mortality in children.

## Figures and Tables

**Table 1:** Age-stratified diagnostic performance of the rapid antigen detection test.

Age Group	n	Sensitivity, % (95% CI)	Specificity, % (95% CI)
<3	42	100.00 (51.01–100.00)	94.74 (82.71–98.54)
3–5	80	100.00 (75.75–100.00)	97.06 (89.90–99.19)
6–12	205	98.25 (90.71–99.69)	95.95 (91.44–98.13)
13–17	62	100.00 (85.13–100.00)	97.50 (87.12–99.56)
Overall	389	98.95 (94.28–99.81)	96.26 (93.43–97.90)

CI, confidence interval. Sensitivity and specificity are calculated using throat culture as the reference standard. The 95% CIs are calculated using the Wilson score method.

**Table 2:** Culture positivity and RADT accuracy by symptom group.

Symptom Group	Total (n)	Culture Positive (n)	RADT Positive (n)	Culture Positivity (%)	RADT Accuracy (%)
Fever only	69	6	6	8.7	100.0
Sore throat only	59	8	9	13.6	98.3
Fever + sore throat	155	68	74	43.9	94.8
Other/unspecified	106	13	16	12.3	97.2

RADT, rapid antigen detection test. “Other/unspecified” includes symptom constellations not categorized above or mixed symptom profiles.

**Table 3:** Multivariable logistic regression analysis: predictors of culture positivity.

Variable	β Coefficient	OR (95% CI)	*p*-Value	Interpretation
Age (years)	0.105	1.11 (1.05–1.18)	<0.001	Positive association
Fever (present)	0.960	2.61 (1.38–4.93)	0.003	Significant predictor
Recent antibiotic use	−0.88	0.42 (0.20–0.90)	0.026	Reduces positivity
Sore throat duration (days)	0.06	1.06 (1.00–1.12)	0.042	Modest positive effect
Tonsillar exudate	1.75	5.75 (2.50–13.24)	<0.001	Strongest predictor
Lymphadenopathy	0.92	2.51 (1.15–5.49)	0.021	Significant predictor

OR indicates odds ratio; CI indicates confidence interval. Multivariable logistic regression model adjusted for age, fever, recent antibiotic use, duration of sore throat, tonsillar exudate, and lymphadenopathy.

**Table 4:** Performance characteristics of RapidFor™ Strep A test compared with throat culture.

RADT	Culture Positive (n)	Culture Negative (n)
Positive	94 (TP)	11 (FP)
Negative	1 (FN)	283 (TN)
Total	95	294

TP indicates true positive; FP, false positive; FN, false negative; TN, true negative.

## Data Availability

The data presented in this study are available upon request from the corresponding author. The data are not publicly available due to privacy and ethical reasons.

## Abbreviations

The following abbreviations are used in this manuscript:

GAS	Group A Streptococcus
RADT	Rapid Antigen Detection Test
PPV	Positive Predictive Value
NPV	Negative Predictive Value
CI	Confidence Interval
ROC	Receiver Operating Characteristic
AUC	Area Under the Curve
OR	Odds Ratio
SD	Standard Deviation
IQR	Interquartile Range
TP	True Positive
FP	False Positive
FN	False Negative
TN	True Negative
WHO	World Health Organization

## References

[1] Walker M.J., Barnett T.C., McArthur J.D., Cole J.N., Gillen C.M., Henningham A., Sriprakash K.S., Sanderson-Smith M.L., Nizet V. (2014). Disease manifestations and pathogenic mechanisms of Group A Streptococcus. Clin. Microbiol. Rev..

[2] Koutouzi F., Tsakris A., Chatzichristou P., Koutouzis E., Daikos G.L., Kirikou E., Petropoulou N., Syriopoulou V., Michos A. (2015). Streptococcus pyogenes emm types and clusters during a 7-year period (2007–2013) in pharyngeal and nonpharyngeal pediatric isolates. J. Clin. Microbiol..

[3] Armstrong G.L., Pinner R.W. (1999). Outpatient visits for infectious diseases in the United States, 1980 through 1996. Arch. Intern. Med..

[4] Carapetis J.R., Steer A.C., Mulholland E.K., Weber M. (2005). The global burden of group A streptococcal diseases. Lancet Infect. Dis..

[5] Bisno A.L., Gerber M.A., Gwaltney J.M., Kaplan E.L., Schwartz R.H., Infectious Diseases Society of America (2002). Practice guidelines for the diagnosis and management of group A streptococcal pharyngitis. Clin. Infect. Dis..

[6] Singh S., Dolan J.G., Centor R.M. (2006). Optimal management of adults with pharyngitis: A multi-criteria decision analysis. BMC Med. Inform. Decis. Mak..

[7] Pelucchi C., Grigoryan L., Galeone C., Esposito S., Huovinen P., Little P., Verheij T. (2012). ESCMID Sore Throat Guideline Group. Guideline for the management of acute sore throat. Clin. Microbiol. Infect..

[8] World Health Organization (2025). WHO Guideline on the Prevention and Diagnosis of Rheumatic Fever and Rheumatic Heart Disease.

[9] Shulman S.T., Bisno A.L., Clegg H.W., Gerber M.A., Kaplan E.L., Lee G., Martin J.M., Van Beneden C. (2012). Clinical practice guideline for the diagnosis and management of Group A streptococcal pharyngitis: 2012 update by the Infectious Diseases Society of America. Clin. Infect. Dis..

[10] Gerber M.A., Shulman S.T. (2004). Rapid diagnosis of pharyngitis caused by group A streptococci. Clin. Microbiol. Rev..

[11] Shaikh N., Leonard E., Martin J.M. (2010). Prevalence of streptococcal pharyngitis and streptococcal carriage in children: A meta-analysis. Pediatrics.

[12] Wi D., Choi S.-H. (2021). Positive rate of tests for Group A Streptococcus and viral features in children with acute pharyngitis. Children.

[13] Kalra M.G., Higgins K.E., Perez E.D. (2016). Common questions about streptococcal pharyngitis. Am. Fam. Physician.

[14] Ashurst J.V., Weiss E., Tristram D., Edgerley-Gibb L. (2025). Streptococcal pharyngitis. StatPearls [Internet].

[15] Taylor A., Morpeth S., Webb R., Taylor S. (2021). The utility of rapid Group A Streptococcus molecular testing compared with throat culture for the diagnosis of Group A streptococcal pharyngitis in a high-incidence rheumatic fever population. J. Clin. Microbiol..

[16] Homme J.H., Greenwood C.S., Cronk L.B., Nyre L.M., Uhl J.R., Weaver A.L., Patel R. (2018). Duration of group A Streptococcus PCR positivity following antibiotic treatment of pharyngitis. Diagn. Microbiol. Infect. Dis..

[17] Tanz R.R., Gerber M.A., Kabat W., Rippe J., Seshadri R., Shulman S.T. (2009). Performance of a rapid antigen-detection test and throat culture in community pediatric offices: Implications for management of pharyngitis. Pediatrics.

[18] Sayğılı N., Bulut E., Deniz R., Dalgıç N., Aktaş E. (2017). Use of Bionexia Strep A Plus Rapid Antigen Test in the identification of Group A beta-hemolytic streptococci in throat swab samples. Türk Mikrobiyol. Cem. Derg..

[19] Altundağ Altun H., Meral T., Türk Arıbaş E. (2015). The specificity and sensitivity results of the rapid antigen test used in the diagnosis of group A beta-hemolytic streptococcal tonsillopharyngitis. Acta Med. Mediterr..

[20] Stewart E.H., Davis B., Clemans-Taylor B.L., Littenberg B., Estrada C.A., Centor R.M. (2014). Rapid antigen group A Streptococcus test to diagnose pharyngitis: A systematic review and meta-analysis. PLoS ONE.

[21] Alp E.E., Dalgıç N., Kına N., Bayraktar B., Öncül A., Sepetci E.A. (2018). Grup A streptokok tonsillofarenjitinde Strep A hızlı antijen testinin önemi: Tek merkez deneyimi. J. Pediatr. Inf..

[22] Sølvik U.Ø., Boija E.E., Ekvall S., Jabbour A., Breivik A.C., Nordin G., Sandberg S. (2021). Performance and user-friendliness of the rapid antigen detection tests QuickVue Dipstick Strep A test and DIAQUICK Strep A Blue Dipstick for pharyngotonsillitis caused by Streptococcus pyogenes in primary health care. Eur. J. Clin. Microbiol. Infect. Dis..

[23] Lean W.L., Arnup S., Danchin M., Steer A.C. (2014). Rapid diagnostic tests for group A streptococcal pharyngitis: A meta-analysis. Pediatrics.

[24] Cohen J.F., Bertille N., Cohen R., Chalumeau M. (2016). Rapid antigen detection test for group A streptococcus in children with pharyngitis. Cochrane Database Syst. Rev..

[25] Macaskill P., Gatsonis C., Deeks J., Harbord R., Takwoingi Y. (2010). Cochrane Handbook for Systematic Reviews of Diagnostic Test Accuracy.

[26] Jo S.A., Ma S.H., Kim S. (2021). Diagnostic impact of clinical manifestations of group A streptococcal pharyngitis. Infect. Chemother..

[27] Zaki Z., Wahab A.A., Ramli R., Esa A., Monoto E.M.M. (2021). Evaluation of rapid antigen detection test for Group A Streptococci pharyngitis among children in an outpatient clinic in Malaysia. Sains Malays..

[28] Gurol Y., Akan H., Izbirak G., Tekkanat Z.T., Gunduz T.S., Hayran O., Yilmaz G. (2010). The sensitivity and the specificity of rapid antigen test in streptococcal upper respiratory tract infections. Int. J. Pediatr. Otorhinolaryngol..

